# Childcare Affordability and Benefits Among Resident Physicians

**DOI:** 10.1001/jamanetworkopen.2025.11089

**Published:** 2025-05-16

**Authors:** Ryan C. L. Brewster, Alex Butler, Katherina Tanson, Shafeeque Kunhiabdullah, Jennifer Kesselheim, Catherine D. Michelson

**Affiliations:** 1 Department of Pediatrics, Boston Children’s Hospital, Boston, Massachusetts; 2 Department of Pediatrics, Boston Medical Center, Boston, Massachusetts; 3 Department of Neonatology, Beth Israel Deaconess Medical Center, Boston, Massachusetts; 4 Department of Pediatric Oncology, Dana-Farber Cancer Institute, Boston, Massachusetts; 5 Department of Pediatrics, Ann and Robert H. Lurie Children’s Hospital of Chicago, Chicago, Illinois

## Abstract

This cross-sectional study evaluates trends in childcare affordability for US resident physicians across all accredited residency programs.

## Introduction

Although residency coincides with peak reproductive years for physicians, extensive training duration, hours, and requirements combined with financial strain can pose formidable barriers to family building.^1^ Previous research has found inadequate supports for resident parents, including access to childcare, but has been limited to qualitative studies or subsets of institutions and specialties.^2^ We aimed to evaluate national trends in childcare affordability for residents and availability of childcare benefits across all US Accreditation Council for Graduate Medical Education (ACGME)–accredited residency programs.

## Methods

Boston Children’s Hospital Institutional Review Board deemed this cross-sectional study exempt from review with a waiver of informed consent because it was not considered human participant research. The STROBE reporting guideline was followed. Childcare benefits–including childcare subsidies and onsite childcare–and salaries for the 2023 to 2024 postgraduate year 1 were obtained for all categorical ACGME-accredited residency programs and sponsoring institutions from the American Medical Association Fellowship and Residency Electronic Interactive Database (eMethods in Supplement 1).^3^ We assumed a 2-partner household with a single infant enrolled in center-based, nonresidential care. The 2023 median annual price for county-level, single infant, center-based childcare was calculated from the US Department of Labor and linked to sponsoring institutions by county.^4^ We used 2023 county-level income estimates from the US Census to approximate a resident partner’s median income.

The Department of Health & Human Services defines affordable childcare as not exceeding 7% of a household’s income. We used this definition to calculate the childcare affordability index by dividing a household’s annual childcare costs by annual gross income (combined income of resident and partner). Lastly, we evaluated trends in inflation-adjusted childcare costs and resident salaries from July 2000 to July 2023 using the Consumer Price Index for All Urban Consumers: Childcare and Nursery School and the 2023 Association of American Medical Colleges Survey of Resident/Fellow Stipends and Benefits.^5^

Factors associated with the childcare affordability index were evaluated with multivariable linear regression, with adjustment for institutional characteristics. Regression covariables were determined a priori and counties within states were clustered to account for spatial correlation. We used the modified Mann-Kendall trend test for temporal trends in childcare costs relative to resident salaries. Statistical analyses were performed in R, version 3.5.2, with a 2-sided *P* < .05 considered significant.

## Results

Among 936 sponsoring institutions analyzed, 520 (56.6%) were community-based with a university affiliation, 393 (42.0%) were in a medium metropolitan area and 294 (31.4%) in an urban area with 1 to 2 associated residency programs (619 [66.1%]) (Table). Using the HHS definition, childcare was unaffordable at 856 institutions (91.6%). The median (IQR) childcare affordability index was 10.3% (8.5%-12.3%). In all, 111 sponsoring institutions (11.9%) offered childcare subsidies and 239 (25.5%) offered onsite childcare. Childcare affordability index was associated with urbanicity (adjusted β, 1.12; 95% CI, 0.29-1.96) and geography (eg, Pacific vs South Atlantic: adjusted β, 3.75; 95% CI, 2.19-5.32) but not with subsidized or onsite childcare availability (Table). From 2000 to 2023, inflation-adjusted childcare costs increased 26.4% (*P* < .001) while resident salaries increased 1.2% (*P* = .03) (Figure).

**Table.** Characteristics of Sponsoring Institutions and Multiple Linear Regression of Associations Between Institution Characteristics and Childcare Affordability Index

Characteristic	Institutions, No. (%) (N = 936)	β (95% CI)	
		Unadjusted	Adjusted
Childcare affordability index			
≥7%	856 (91.6)	NA	NA
<7%	80 (8.6)	NA	NA
Subsidized childcare	111 (11.9)	0.07 (−0.47 to 0.62)	−0.11 (−0.53 to 0.31)
On-site childcare	239 (25.5)	−0.02 (−0.42 to 0.38)	−0.21 (−0.69 to 0.27)
US region^a^			
East North Central (Illinois, Indiana, Michigan, Ohio, Wisconsin)	163 (17.4)	2.27 (1.81 to 2.73)	2.0 (0.81 to 3.19)
East South Central (Alabama, Kentucky, Mississippi, Tennessee)	41 (4.4)	−1.41 (−2.14 to −0.69)	−0.96 (−2.48 to 0.57)
Mid-Atlantic (New Jersey, New York, Pennsylvania)	194 (20.7)	2.8 (2.36 to 3.24)	2.5 (0.37 to 4.63)
Mountain (Arizona, Colorado, Idaho, Montana, New Mexico, Nevada, Utah, Wyoming)	38 (4.1)	0.17 (−0.58 to 0.92)	0.04 (−1.39 to 1.47)
New England (Connecticut, Maine, Massachusetts, New Hampshire, Rhode Island, Vermont)	57 (6.1)	3.71 (3.07 to 4.35)	3.75 (2.19 to 5.32)
Pacific (Alaska, California, Hawaii, Oregon, Washington)	129 (13.8)	3.88 (3.39 to 4.37)	3.42 (2.37 to 4.46)
South Atlantic (District of Columbia, Delaware, Florida, Georgia, Maryland, North Carolina, South Carolina, Virginia, West Virginia)	169 (18.1)	[Reference]	[Reference]
West North Central (Iowa, Kansas, Minnesota, Missouri, Nebraska, North Dakota, South Dakota)	56 (6.0)	0.83 (0.19 to 1.48)	0.87 (−0.79 to 2.54)
West South Central (Arkansas, Louisiana, Oklahoma, Texas)	89 (9.5)	−1.16 (−1.71 to −0.61)	−1.09 (−2.11 to −0.07)
Institution type^b^			
Community-based	178 (19.0)	[Reference]	[Reference]
Community-based university-affiliated	520 (55.6)	0.77 (0.31 to 1.23)	0.02 (−0.29 to 0.32)
University-based	235 (25.1)	0.64 (0.11 to 1.17)	−0.29 (−0.93 to 0.35)
Military-based	2 (0.3%)	4.61 (0.79 to 8.43)	2.08 (−0.16 to 4.31)
Residency programs per institution			
1-2	619 (66.1)	[Reference]	[Reference]
3-4	110 (11.8)	0.42 (−0.14 to 0.98)	−0.05 (−0.45 to 0.36)
≥5	207 (22.1)	0.33 (−0.11 to 0.76)	0.34 (−0.31 to 0.98)
Rural-urban status^c^			
Medium metropolitan	393 (42.0)	[Reference]	[Reference]
Rural	28 (3.0)	−2.81 (−3.77 to −1.85)	−2.12 (−2.68 to −1.57)
Small metropolitan	221 (23.6)	−1.23 (−1.65 to −0.82)	−0.78 (−1.19 to −0.37)
Urban	294 (31.4)	1.53 (1.15-1.91)	1.12 (0.29 to 1.96)

Abbreviations: NA, not applicable.

^a^ Geographic regions are defined by the US Census Bureau.

^b^ The American Medical Association Fellowship and Residency Electronic Interactive Database classifies sponsoring institutions into different hospital types. Community-based programs do not take place in a university academic medical center or hospital with a medical school affiliation. Community-based university-affiliated programs take place in a community hospital that is affiliated with an academic medical center but is not a primary affiliate or is geographically separate from the academic medical center. University-based programs take place in a hospital that serves as a primary affiliate of the medical school.

^c^ The National Center for Health Statistics Rural-Urban Classification Scheme assigns counties to different categories based on population size, per the 2013 US Census: Large metropolitan (>1 million), medium metropolitan (250 000-999 999), small metropolitan (50 000-249 999), and rural (<50 000).

**Figure.**
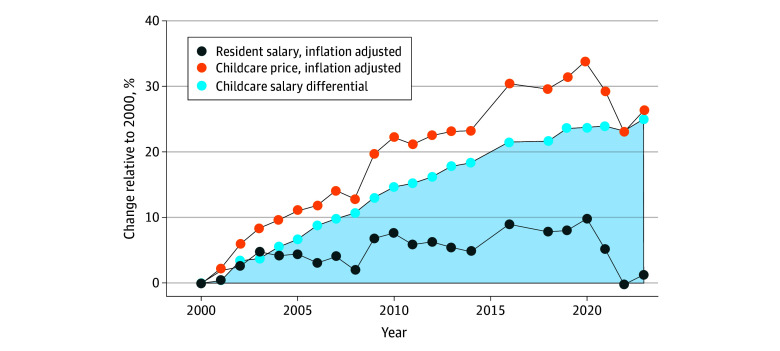
Childcare Prices and Postgraduate Year 1 (PGY-1) Resident Salaries From 2000 to 2023 Childcare prices and PGY-1 salaries were inflation-adjusted with the Consumer Price Index for All Urban Consumers US city average.

## Discussion

Childcare has become increasingly unaffordable for residents, with a widening disparity between resident salaries and childcare costs over the last 20 years. Meanwhile, a minority of sponsoring institutions offer childcare subsidies or onsite childcare.

These findings extend prior research^1,2^ emphasizing the structural and cultural challenges to effective family building. While rising childcare costs are ubiquitous, residents may be uniquely disadvantaged due to long, inflexible duty hours, inconsistent parental leave and benefits, and lack of negotiated benefits at hire. Childcare may compound with other cost of living considerations, such as housing, suggesting financial strain for many trainees, especially in coastal, metropolitan centers.^6^

Study limitations include use of population-level data sources, which prevented adjustment for different family arrangements (eg, single-parent households) and a partner’s actual income. Ultimately, multilevel advocacy is needed for national childcare reform, strengthening of institutional benefits, and reformulation of graduate medical education reimbursements to support family building among physician parents.

## Notes

Supplement 1.
eMethods.

eReferences

Supplement 2.
Data Sharing Statement
