# Supplementary material for: Childcare Affordability and Benefits Among Resident Physicians
Source: JAMA Netw Open. 2025 May 16;8(5):e2511089. doi: 10.1001/jamanetworkopen.2025.11089 (PMC12084837; doi:10.1001/jamanetworkopen.2025.11089)
Supplement: Supplement 1. — eMethods. eReferences [file jamanetwopen-e2511089-s001.pdf]

Supplemental Online Content

Brewster RCL, Butler A, Tanson K, Kunhiabdullah S, Kesselheim J, Michelson CD. Childcare affordability and benefits among resident physicians. *JAMA Netw Open.* 2025;8(5): doi: 10.1001/jamanetworkopen.2025.11089

This supplemental material has been provided by the authors to give readers additional information about their work.

eMethods.
eReferences

© 2025 Brewster RCL et al. *JAMA Network Open*.

eMethods

Fellowship and Residency Electronic Interactive Database (FREIDA)

FREIDA is a freely available database curated by the American Medical Association (AMA) of residency and fellowship programs accredited by the Accreditation Council for Graduate Medical Education (ACGME). Program listings include an overview of the training curriculum and compensation information, among other details. The National Graduate Medical Education Census, jointly administered by the AMA and Association of American Medical Colleges (AAMC), collects institutional characteristics from program directors and administrators each year. Responses to benefit information are dichotomous (yes/no); if no response is entered, the benefit is omitted from the final published listing. Of note, there were no missing data among the sponsoring institutions included for analysis.

National Database of Childcare Prices

Developed by the US Department of Labor, the National Database of Childcare Prices (NDCP) provides a comprehensive reference for county-level childcare prices by provider type (center-based, home-based), age of children (infant, toddler, pre-school, school-age) from 2008-2023.[1] Median and 75th percentile estimates are calculated from market rate surveys (MRS) reported by states to the US Department of Health and Human Services to inform state reimbursement rates for childcare services.

Resident Household Structure and Income

To our knowledge, the family composition of resident physicians – including partnership status, number and ages of children, and types of childcare – has not been previously described. In the absence of empirical data, our analysis assumed that a household comprised a resident and partner with a shared income and single infant-aged child who received care in a non-residential setting (center-based care). To approximate a resident partner’s income, we used estimates of 2023 per capita personal income by county from the U.S. Census Bureau.[2]

Reconciling Residency Program and Sponsoring institution Characteristics

In this analysis, the term *residency program* is defined as an accredited medical residency program at an approved, qualifying institution of which some portion of the education occurs as direct instruction. The term *sponsoring institution* is defined as an entity that oversees, supports, and administers one or more ACGME-accredited residency/fellowship programs.

Resident salaries and benefits are standardized within a sponsoring institution, regardless of specialty, as stipulated by direct graduate medical education (GME) payments. To reconcile differences in reported salary within sponsoring institutions, we

selected the highest salary reported by a sponsoring institution and applied that value to all programs within that institution. Similarly, if at least one specialty provided a particular benefit within a sponsoring institution, it was assumed that all specialties would receive the same benefit. We manually corroborated salary and benefit packages with program website and recruitment materials, where available.

Consumer Price Index

Consumer Price Index (CPI), as defined by the Bureau of Labor and Statistics, measures the monthly change in prices paid by U.S. consumers.[3] This number is a weighted average of prices for a collection of goods and services representative of aggregate U.S. consumer spending. To estimate changes in childcare prices over time, we used a component of the CPI for Childcare and Nursery School (Also referred to as Day Care and Preschool), which encompasses costs for preschool-age children, pre-K educational programming, and "individuals whose occupation is to regularly care for pre-elementary school children."[3]

eReferences

1. United States Department of Labor. National Database of Childcare Prices. https://www.dol.gov/agencies/wb/topics/featured-childcare
2. United States Census Bureau. *Population and Housing Unit Estimates Tables*. https://www.census.gov/programs-surveys/popest/data/tables.html
3. U.S. Bureau of Labor Statistics. *Consumer Price Index*. https://www.bls.gov/cpi/additional-resources/entry-level-item-descriptions.htm#:~:text=tuition%20and%20fees.-,Education%20and%20Communication,-EB031
