# Supplementary material for: Childcare Affordability and Benefits Among Resident Physicians
Source: JAMA Netw Open. 2025 May 16;8(5):e2511089. doi: 10.1001/jamanetworkopen.2025.11089 (PMC12084837; doi:10.1001/jamanetworkopen.2025.11089)
Supplement: Supplement 2. — Data Sharing Statement [file jamanetwopen-e2511089-s002.pdf]

Data Sharing Statement

Brewster. Childcare Affordability and Benefits Among Resident Physicians. *JAMA Netw Open*. Published May 16, 2025. doi:10.1001/jamanetworkopen.2025.11089

Data
Data available: No
